# Current status of robot‐assisted surgery in the clinical application of trauma orthopedics in China: A systematic review

**DOI:** 10.1002/hsr2.930

**Published:** 2022-11-08

**Authors:** Ding Xu, Weigang Lou, Ming Li, Jingwei Xiao, Hongbao Wu, Jianming Chen

**Affiliations:** ^1^ Department of Orthopedic Trauma Surgery Ningbo No. 6 Hospital Ningbo China; ^2^ Department of Surgery Ningbo University Medical College Ningbo China

**Keywords:** China, clinical application, robot‐assisted surgery, trauma orthopedics

## Abstract

**Background and Aims:**

To elaborate on the development and characteristics of trauma orthopedic robots and their real curative effect in a clinical application through the collection and analysis of relevant literature and reported clinical results.

**Method:**

We conducted the Embase, ScienceDirect, Pubmed, Medline, Wanfang, CNKI, and VIP search of the literature on robotic‐assisted surgery in trauma orthopedics in China. We combined search terms with “robotic surgery/artificial intelligence surgery/navigation surgery,” “trauma/trauma orthopedics,” and “China/Chinese.” The exclusion criteria were: (1) articles in languages other than English or Chinese, (2) articles focused on other topics other than robotic‐assisted surgery in trauma orthopedics of China, (3) article types were not clinical studies (reviews, basic research, etc.), and (4) articles were not included in the Chinese core journals or science citation index. Authors, type of surgery, robot type, and clinical research results were recorded and analyzed.

**Results:**

There were three categories of surgical robots in the clinical application of trauma orthopedics (TiRobot, electromagnetic navigation surgical robots, and small medical robots developed by Beijing Jishuitan Hospital). In terms of blood loss, the fluoroscopy time, and fluoroscopy frequency, most studies found that the robot group was significantly better than the traditional group.

**Conclusions:**

Robot‐assisted surgery has obvious advantages in accuracy, stability, and reducing intraoperative radiation exposure, but there is no final conclusion about functional recovery.

## 1 INTRODUCTION

The clinical application of robot‐assisted surgery has become possible with the improvement of robot technology (computer control technology, detection technology, image processing technology, multimedia and information network technology, man–machine interface technology, and mechanical and electronic technology) and the rapid development of minimally invasive surgery.^1^ As early as the 1980s, surgical robots came into being. In 1985, robots were first used in brain surgery.^2^ Because of the advantages of good stability, flexible operation, and accurate movement, surgical robots were more and more used in clinical treatment. In 1992, the first robot system (Robodoc) in the world was applied to hip replacement surgery, creating a precedent for the application of surgical robots in orthopedics.^3^, ^4^ In recent years, the types of orthopedic surgical robots were becoming more and more complete, and the application scope and scene were also gradually expanding. The robot systems such as Robodoc (Curexo Technology Company), SpineAssist (Mazor Robotic Company), Stryker‐Nav (Stryker Corporation), and Renaissance (Mazor Robotic Company) had developed rapidly and had been successfully used in hip and knee arthroplasty, pedicle screw placement, and other operations.^5^, ^6^ Compared with joint surgery and spine surgery, there were few surgical robots used in trauma orthopedics.^7^ However, this situation had changed because of the rapid development of image processing technology and the improvement of navigation tracers in recent years.^8^ Surgical robot was reported to be applied to the precise positioning and nail placement in orthopedic trauma surgery to improve surgical accuracy and reduce surgical injury.^7^ Especially in China, the application research of computer‐assisted navigation surgery and medical robots in trauma orthopedics was funded by national scientific research projects in 2001. After that, research reports on the clinical application of robots in trauma orthopedics appeared one after another.^9^ However, there was a lack of systematic review and summary on the clinical application of robots in trauma orthopedics in China. The purpose of this paper was to elaborate on the development and characteristics of trauma orthopedic robots in China and their real curative effect in a clinical application through the collection and analysis of relevant literature and reported clinical results.

## 2 METHODS

This systematic review was conducted in accordance with the preferred reporting items for systematic reviews guidelines.

### 2.1 Search strategy

Publications related to robotic‐assisted surgery in trauma orthopedics in China were identified through a computerized literature search. We conducted an Embase, ScienceDirect, Pubmed, Medline, Wanfang, CNKI, and VIP search of the literature on robotic‐assisted surgery in trauma orthopedics in China. We combined search terms with “robotic surgery/artificial intelligence surgery/navigation surgery,” “trauma/trauma orthopedics,” and “China/Chinese.” All electronic searches were conducted within a day of March 7, 2022, to avoid changes in citation rates as much as possible.

### 2.2 Study selection

Two independent reviewers (Ding Xu and Weigang Lou) reviewed all articles by reading the abstracts. When it was necessary, the full texts were acquired from Embase, Medline, Pubmed, ScienceDirect, Wanfang, CNKI, or VIP. The CNKI, Wanfang, and VIP were the three databases of Chinese periodicals. Only studies focusing on robotic‐assisted surgery in trauma orthopedics in China as the main topic were included. The exclusion criteria were: (1) articles in languages other than English or Chinese, (2) articles focused on other topics other than robotic‐assisted surgery in trauma orthopedics of China, (3) article types were not clinical studies (reviews, basic research, etc.), (4) articles were not included in the Chinese core journals or science citation index. Chinese core journals included the core journal criterion of the Institute of Scientific and Technical Information of China, the Chinese core journal criterion of Peking University, and the Chinese Science Citation Database. Any disagreements between the two reviewers were resolved by discussion with the third reviewer (Ming Li).

### 2.3 Data extraction

Two authors (Jingwei Xiao and Hongbao Wu) independently extracted data with a structured data collection form. Discrepancies were resolved by discussion with the senior investigator (Jianming Chen). The following information was sought from each article: (1) authors (first author), (2) publication year, (3) type of disease, (4) type of surgery, (5) robot type, (6) published journal, and (7) clinical research results (follow‐up time, blood loss, operating time, fluoroscopy frequency, functional score, internal fixation placement, fracture healing, etc.). The level of evidence for clinical studies was also determined by an assessment based on the level of evidence from the Oxford Centre for Evidence‐Based Medicine (OCEBM).^10^ There was 100% agreement between the two authors about the level of evidence.

### 2.4 Evaluating the included studies

Based on included study design, research setting, and goals, the selected articles were all clinical studies, mainly divided into case reports, case series, retrospective cohort studies, prospective cohort studies, and randomized control trials (RCTs).

### 2.5 Methodological quality assessment

Levels of evidence were assessed using the OCEBM framework. The quality of clinical studies was independently assessed by two authors (Jingwei Xiao and Hongbao Wu) using the methodological index for non‐randomized studies tool.^11^ The randomized controlled trials were scored using the revised Cochrane risk‐of‐bias tool (RoB 2).^12^ The risk of bias in the following seven areas was assessed as “low,” “high,” or “unclear”: sequence generation; allocation concealment; whether the participants, personnel, and results were evaluated by blind method; incomplete outcome data; selective reporting; and other sources of bias (if needed).

### 2.6 Analysis

A data collection table in Microsoft Excel was designed by one author (Weigang Lou) to display the information extracted from each eligible study. Owing to heterogeneity in study design, participants, interventions, and outcome measures, a quantitative meta‐analysis was not appropriate.

## 3 RESULTS

**Figure 1:**
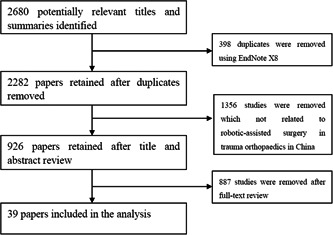
Flow diagram for selection and inclusion of studies

A total of 2680 articles were included after the initial search. Among them, there were 581 articles from Wanfang. CNKI and VIP were 226 and 354 articles, respectively. The number of articles from English databases was 570 (Pubmed), 320 (Embase), 270 (Medline), and 359 (ScienceDirect). A total of 398 duplicates were removed using EndNote X8 (Thomson Reuters) leaving 2282 studies. The titles and abstracts were reviewed to eliminate 1356 studies which not related to robotic‐assisted surgery in trauma orthopedics in China. We then reviewed the full text of the remaining studies to eliminate 887 studies that were not clinical studies. Ultimately, 39 papers were included in the analysis (Figure 1). Fifteen of them were in English and 24 were in Chinese.

### 3.1 Study characteristics

Regarding the time of publication, the earliest year could be traced back to 2005. The year with the most published articles was 2019 (*n* = 10). There were eight kinds of diseases involved, the most was pelvic acetabular injury (*n* = 21), followed by femoral neck fracture (*n* = 7). Others involved tibial shaft fracture, Lisfranc injury, tibial plateau fracture and femoral trochanter fracture, etc. The internal fixation instruments involved in the operation were mainly hollow lag screws (*n* = 29), and others included proximal femoral intramedullary nails, tibial intramedullary nails, and countersunk compression screws. There were three categories of surgical robots in the clinical application of trauma orthopedics. The first category was the TiRobot system (Tinavi Medical Technologies Company). A total of 33 articles mentioned this type of surgical robot, and another article used the previous generation product (GD2000) of this robot system. The other two categories were electromagnetic navigation surgical robots produced by Weigao company (*n* = 1) and small medical robots jointly developed by Beijing Jishuitan Hospital and Beijing University of Aeronautics and Astronautics (*n* = 3) (Table 1). A total of 18 journals had reported on surgical robots in the clinical application of trauma orthopedics in China, 13 articles in Chinese and 5 articles in English. *Chinese Journal of Orthopedic Trauma* was the most involved Chinese journal in terms of articles published (*n* = 6). At the same time, the most involved English journal was *Orthopedic Surgery*. There were five types of clinical studies. Among them, the most type was a retrospective cohort study, with a total of 18 articles. The second was the case series (*n* = 17). The rest were prospective controlled studies, RCTs, and case reports.

**Table 1:** The categories of surgical robots in the clinical application of trauma orthopedics

	TiRobot system	GD2000	Electromagnetic navigation surgical robots	Small medical robots
Manufacturer	Tinavi Medical Technologies Company	Tinavi Medical Technologies Company	Weigao company	Beijing Jishuitan Hospital and Beijing University of Aeronautics and Astronautics
Year	2016	2013	2019	2005
Quantity	33	1	1	3
Description	It is used in spinal and traumatic orthopedic surgery to significantly reduce intraoperative radiation.	The previous generation product of the TiRobot system	Surgical robot with magnetic navigation system	Small surgical robot applied to closed reduction and internal fixation of tibial fractures with interlocking intramedullary nails

*Note*: The categories of surgical robots in the clinical application of trauma orthopedics.

Our research showed that 84.6% of the included articles used this type of robot, which was the main model of trauma orthopedic robot undoubtedly.

### 3.2 Clinical research outcomes

#### 3.2.1 Operating time

Because of the different operation sites, the operation duration was different. Among the 20 cohort studies, 10 retrospective cohort studies showed that the robot operation time was significantly shorter than that of the traditional group, which was statistically significant. However, five retrospective cohort studies showed no significant difference between the two groups, including a study comparing robots with O‐arm navigation.^13^ Three retrospective cohort studies did not involve the study of operation time (Table 2). It was important to note that both an RCT study and a prospective cohort study showed no significant difference between the two groups regarding the operating time.^14^, ^15^

**Table 2:** Various clinical data sheets of all articles

Number	Author	Language	Year	Surgical site	Operating time (min)	blood loss (ml)	fluoroscopy frequency (times)	Fluoroscopy time (s)	functional score	fracture healing	Clinical study category	Level of evidence
1	Zeng TY	Chinese	2015	Femoral neck	N/A	N/A	Robot: 23.1 Convention: 52.6	Robot: 15.7 Convention: 29.8	N/A	N/A	Retrospective cohort studies	III
2	Tan Z	Chinese	2019	Intertrochanteric	Robot: 67.50 ± 9.79 Convention: 78.94 ± 16.85 *p* = 0.002	Robot: 91.07 ± 14.23 Convention: 115.16 ± 30.54 *p* ˂ 0.001	Robot: 10.36 ± 0.68 Convention: 13.32 ± 1.64 *p* ˂ 0.001	N/A	Harris score Robot: 86.857 ± 5.936 Convention: 83.258 ± 6.683 *p* ˂ 0.05	Robot: 100% Convention: 100% *p* > 0.05	Retrospective cohort studies	III
3	Gu SL	Chinese	2020	Pelvis and acetabulum	Robot: 74.8 ± 10.7 Convention: 85. 4 ± 11. 4 *p* = 0.082	Robot: 5–15 Convention: 15–80	Robot: 23‐41 Convention: 36–66 *p* = 0.014	N/A	N/A	Robot: 100% Convention: 100% *p* > 0.05	Retrospective cohort studies	III
4	Wang JF	Chinese	2008	Tibia	Robot: 78 (range 52–129)	N/A	N/A	Robot: 13.3 (range 10.6–22)	N/A	N/A	Case series	IV
5	Hu PY	Chinese	2020	Pelvis	Robot: 120	Robot: 20	N/A	N/A	N/A	N/A	Case report	IV
6	Wang JQ	Chinese	2005	Tibia	Robot: 77 (range 46–110)	N/A	Robot: 19	N/A	N/A	N/A	Case series	IV
7	Lin WD	Chinese	2021	Pelvis	Robot: 120 ± 22.2 Convention: 224.4 ± 258. 4 *p* ˂ 0.001	Robot: 10.60 ± 1.66 Convention: 13.23 ± 3.99 *p* = 0.002	Robot: 7.44 ± 1.45 Convention: 23.23 ± 5.81 *p* ˂ 0.001	N/A	Braden score Robot: 13.72 ± 1.21 Convention: 11.26 ± 0.82 *p* ˂ 0.001	N/A	Retrospective cohort studies	III
8	Zhao CP	Chinese	2020	Acetabulum	Robot: 198 (range 60–510)	Robot: 298.7 (range 20–2000)	N/A	N/A	Majeed score Robot: 85.4 (60–100)	N/A	Case series	IV
9	Wang G	Chinese	2020	Pelvis	Robot: 14.9/per screw	N/A	Robot: 4.7/per screw	N/A	N/A	Robot: 100%	Case series	IV
10	Liu HS	Chinese	2019	Pelvis	Robot: 106	Robot: 20.1 ± 4.7	Robot: 29.2 ± 10.5	Robot: 6–42	Majeed score Robot: 89.4	N/A	Case series	IV
11	Li CH	Chinese	2021	Pelvis	Robot: 50.2 ± 13.2 Convention: 80.5 ± 16.1 *p* ˂ 0.001	N/A	N/A	Robot: 7.2 ± 1.5 Convention: 25.7 ± 7.6 *p* ˂ 0.001	Majeed score Robot: 97.9 Convention: 79.2 *p* ˂ 0.001	N/A	Retrospective cohort studies	III
12	Yang CZ	Chinese	2021	Pelvis	Robot: 52.52 ± 15.14 O‐arm X: 53.86 ± 15.06 *p* > 0.05	N/A	Robot:0.34 ± 0.06/per screw O⁃arm X: 1.56 ± 0.02/per screw *p* ˂ 0.05	Robot: 6.80 ± 3.20 O‐arm X: 7.36 ± 2.63 *p* > 0.05	Majeed score Robot: 76‐95 O⁃arm X: 55‐87 *p* > 0.05	Robot: 100% O‐arm X: 100% *p* > 0.05	Retrospective cohort studies	III
13	Tong W	Chinese	2016	Femoral neck	Robot: 79.7 ± 15.7 Convention: 74.1 ± 14.9 *p* > 0.05	Robot: 8.0 ± 3.4 Convention: 10.0 ± 2.4 *p* ˂ 0.05	N/A	Robot: 14.0 ± 4.5 Convention: 21.0 ± 5.4 *p* ˂ 0.001	Harris score Robot: 87.1 ± 3.7 Convention: 79.3 ± 4.7 *p* ˂ 0.001	Robot: 100% Convention: 88.9% *p* > 0.05	Retrospective cohort studies	III
14	Fu XF	Chinese	2020	Tibia	Robot: 91.67 ± 6.23 Convention: 92.33 ± 4.45 *p* > 0.05	Robot: 36.00 ± 2.20 Convention: 52.00 ± 5.00 *p* ˂ 0.05	Robot: 11.00 ± 0.75 Convention: 20.22 ± 1.33 *p* ˂ 0.05	N/A	Rasmussen score Robot: 28.89 ± 0.31 Convention: 27.22 ± 0.62 *p* ˂ 0.05	Robot: 100% Convention: 96.1% *p* > 0.05	Retrospective cohort studies	III
15	Li LF	Chinese	2018	Foot	Robot: 50.16 ± 19.32	Robot: 15.37 ± 6.71	Robot: 8.21 ± 3.02	N/A	AOFAS score Robot: 87.32 ± 10.37	N/A	Case series	IV
16	Liu HS	Chinese	2017	Pelvis	Robot: 120	Robot: 50	Robot: 30	Robot: 18	Majeed score Robot: 91	N/A	Case report	IV
17	Zhao CP	Chinese	2017	Pelvis and acetabulum	Robot: 43.86 ± 49.06 Convention: 29.00 ± 12.14 *p* > 0.05	N/A	N/A	Robot: 7.36 ± 2.63 Convention: 41.80 ± 13.99 *p* ˂ 0.001	N/A	N/A	Retrospective cohort studies	III
18	Xiong W	Chinese	2021	Acetabulum	Robot: 78 ± 12	Robot: 161. 3 ± 21. 1	N/A	N/A	Majeed score Robot: 85. 1 ± 1. 9	Robot: 100%	Case series	IV
19	Zhao Y	Chinese	2018	Pelvis	Robot: 15.9/per screw	Robot: <1/per screw	N/A	N/A	VAS score Robot: 1.8	N/A	Case series	IV
20	Long T	Chinese	2019	Pelvis	Robot: 25‐45	Robot: 10–80	Robot: 4–15	Robot: 192–390	N/A	Robot: 100%	Case series	IV
21	Wang JQ	Chinese	2017	Pelvis	Robot: 17/per screw	N/A	N/A	N/A	N/A	Robot: 100%	Case series	IV
22	Shi WX	Chinese	2017	Femoral neck	Robot: 92.2 ± 6.7	Robot: 101 ± 21	N/A	Robot: 16.1 ± 4.1	Harris score Robot: 86. 9 ± 5. 1	Robot: 100%	Case series	IV
23	Han W	English	2022	Pelvis	N/A	N/A	N/A	Robot: 8.06 ± 3.54 s/per screw Convention: 27.27 ± 8.82 s/per screw *p* ˂ 0.001	N/A	N/A	Retrospective cohort studies	III
24	Hu PY	Chinese	2020	Pelvis	Robot: 120	Robot: 20	N/A	N/A	N/A	N/A	Case report	IV
25	Wang JQ	Chinese	2006	Tibia	Robot: 144	N/A	N/A	N/A	N/A	N/A	case series	IV
26	Du W	English	2020	Pelvis	Robot: 112.9 ± 16.8	Robot: 105.9 ± 20.6	N/A	N/A	Majeed score Robot: 91.8 ± 3.8	N/A	Case series	IV
27	Duan SJ	English	2019	Femoral neck	Robot: 62.6 ± 8.7 Convention 72.4 ± 10.3 *p* > 0.05	Robot: 8.2 ± 5.3 Convention: 36.4 ± 12.5 *p* ˂ 0.05	Robot: 26.5 ± 7.4 Convention: 51.3 ± 9.4 *p* ˂ 0.05	N/A	Harris score Robot: 88.2 ± 3.6 Convention: 87.3 ± 4.7 *p* > 0.05	Robot: 88.4% Convention: 82.6% *p* > 0.05	Prospective cohort studies	II
28	He M	English	2019	Femoral neck	N/A	N/A	N/A	Robot: 5.7 s/per screw Convention: 14.14 s/per screw *p* ˂ 0.001	Harris score Robot: 85.2 Convention: 83.45 *p* > 0.05	N/A	Retrospective cohort studies	III
29	Lan H	English	2019	Intertrochanteric	Robot: 65.44 ± 8.01 Convention 77.50 ± 16.64 *p* = 0.002	Robot: 90.8 ± 14.98 Convention: 118.46 ± 32.21 *p* ˂ 0.001	Robot: 10.28 ± 0.61 Convention: 13.23 ± 0.75 *p* ˂ 0.001	N/A	Harris score Robot: 86.68 ± 6.23 Convention: 82.69 ± 6.85 *p* ˂ 0.05	Robot: 100% Convention: 100% *p* > 0.05	Retrospective cohort studies	III
30	Liu HS	English	2018	Pelvis	Robot: 65.4 ± 10.9 Convention: 86.7 ± 14.7 *p* ˂ 0.05	Robot: 35.0 ± 7.2 Convention: 46.2 ± 9.3 *p* ˂ 0.05	Robot: 29.2 ± 7.6 Convention: 52.3 ± 12.4 *p* ˂ 0.05	N/A	Majeed score Robot: 86.4 ± 7.2 Convention: 84.3 ± 10.3 *p* > 0.05	Robot: 100% Convention: 100% *p* > 0.05	Retrospective cohort studies	III
31	Liu HS	English	2019	Pelvis	Robot: 175 (35–280)	Robot: 35.2 ± 3.6 (5–50)	Robot: 29.1 (9–63)	Robot: 6.1 s/per screw	Majeed score Robot: 92.4	Robot: 97.6%	Case series	IV
32	Liu ZJ	English	2021	Pelvis	Robot: 17.0 ± 3.0 Convention: 34.5 ± 10.6 *p* ˂ 0.001	N/A	Robot: 14.8 ± 2.2 Convention: 28.2 ± 5.8 *p* ˂ 0.001	N/A	Majeed score Robot: 86.2 ± 5.7 Convention: 80.1 ± 7.6 *p* ˂ 0.05	Robot: 94.4% Convention: 66.7% *p* ˂ 0.05	Retrospective cohort studies	III
33	Long T	English	2019	Pelvis	Robot: 33.25 ± 6.46 Convention: 63.55 ± 6.62 *p* ˂ 0.001	Robot: 33.89 ± 16.4 Convention: 43.04 ± 12.34 *p* ˂ 0.001	Robot: 8.49 ± 2.37 Convention: 18.67 ± 4.18 *p* ˂ 0.001	Robot: 352.8 ± 77.4 Convention: 663 ± 178.8 *p* ˂ 0.001	Majeed score *p* > 0.05	Robot: 100% Convention: 100% *p* > 0.05	Retrospective cohort studies	III
34	Luo J	English	2020	Femoral Head	Robot: 46.99 ± 4.94 Convention: 55.01 ± 6.19 *p* ˂ 0.001	Robot: 20.62 ± 2.52 Convention: 52.72 ± 3.39 *p* ˂ 0.001	Robot: 10.50 ± 1.78 Convention: 17.91 ± 2.20 *p* ˂ 0.001	N/A	Harris score Robot: 69.53 ± 7.51 Convention: 68.38 ± 7.26 *p* > 0.05	N/A	Retrospective cohort studies	III
35	Wang JQ	English	2017	Pelvis	Robot: 150.0 (75.0–230.0) Convention: 104.0 (60.0–154.0) *p* > 0.05	N/A	N/A	N/A	N/A	N/A	RCT	I
36	Wang XD	English	2019	Femoral neck	Robot: 65.70 ± 9.87 Convention: 73.74 ± 9.78 *p* ˂ 0.001	Robot: 15.25 ± 6.21 Convention: 25.51 ± 6.97 *p* ˂ 0.001	Robot: 13.67 ± 4.39 Convention: 17.09 ± 4.02 *p* ˂ 0.001	N/A	Harris score Robot: 86.86 ± 4.74 Convention: 83.08 ± 5.44 *p* ˂ 0.05	Robot: 100% Convention: 100% *p* > 0.05	Retrospective cohort studies	III
37	Zhu ZD	English	2021	Femoral neck	Robot: 83.3 ± 31.2 Convention: 44.1 ± 14.8 *p* ˂ 0.001	Robot: 11.3 ± 7.3 Convention: 51.6 ± 40.4 *p* ˂ 0.001	Robot: 40.1 ± 28.5 Convention: 38.6 ± 21.0 *p* > 0.05	N/A	Harris score Robot: 93.2 ± 10.3 Convention: 88.4 ± 11.9 *p* > 0.05	Robot: 100% Convention: 92.7% *p* > 0.05	Retrospective cohort studies	III
38	Guo Y	English	2020	Wrist	N/A	N/A	N/A	N/A	N/A	Robot: 100%	Case series	IV
39	Liu B	English	2019	Wrist	Robot: 40 (27–56).	N/A	N/A	N/A	Mayo score Robot: 96	Robot: 100%	Case series	IV

*Note*: Various clinical datasheets of all articles.

Abbreviations: AOFAS, American Orthopedic Foot and Ankle Society Score; N/A, not applicable; RCT, randomized control trial; VAS, visual analog scale.

#### 3.2.2 Blood loss

A total of 23 articles mentioned intraoperative blood loss. All cohort studies (11 articles) showed that the blood loss in the robot group was significantly less than that in the traditional group, which was statistically significant. The 11 cohort studies included 10 retrospective cohort studies and 1 prospective cohort study (Table 2).

#### 3.2.3 Fluoroscopy frequency

The study of fluoroscopy frequency was mentioned in 21 articles. One retrospective cohort study showed that there was no significant difference in fluoroscopy frequency between the robot group and the traditional group.^16^ However, 12 retrospective cohort studies showed statistical differences in fluoroscopy frequency between the two groups, including 1 study comparing robots with O‐arm navigation.^13^ At the same time, one prospective cohort study reached the same conclusion.^15^

#### 3.2.4 Fluoroscopy time

The research on fluoroscopy time was less mentioned, and a total of 14 articles were involved. There were eight cohort studies among them, all of which were retrospective cohort studies. Compared with the traditional group, the fluoroscopy time of the robot group was significantly reduced, which was statistically significant. Only one study suggested that there was no difference in fluoroscopy time, but this study was compared between the group robot and O‐arm navigation.^13^

#### 3.2.5 Functional score

A total of 24 articles discussed functional scores, of which 15 were cohort studies. Among the 15 articles, 8 articles believed that there were significant differences in functional scores between the robot group and the traditional group. However, seven articles showed no statistical difference between the two groups, including a prospective cohort study.^15^

#### 3.2.6 Fracture healing

A total of 19 articles described the situation of fracture healing. Twelve of them were cohort studies. Only 1 of the 12 articles considered that there was a statistical difference in fracture healing between the robot group and the traditional group.^17^ However, the robot group in this study performed bone for pelvic fractures, so the results will be disturbed by the bone grafting operation.

## 4 DISCUSSION

Orthopedic precision treatment technology has become one of the main development directions of surgery in the 21st century.^18^ In particular, the surgical robot as the representative of this technology has become a research hotspot in the field of medicine and robot.^19^ The surgical robot can achieve accurate spatial positioning and stable path navigation, which greatly increases the safety of surgery and reduces the risk and the trauma of surgery.^20^ Especially in the field of orthopedics, robots can replace doctors to complete necessary work in the radiation environment and extend doctors' observation and operation ability. Orthopedic surgical robots in European and American countries have developed earlier and faster, and the types of robots are more comprehensive. Robot systems mainly include Acrobot robot system (Stryker Corporation), Mako plasty robot system (Stryker Corporation), Robodoc robot system (Curexo Technology Company), and SpineAssist robot system (Mazor Robotic Company).^5^, ^6^ Compared with the orthopedic surgical robots in Europe and the United States, the research and development of orthopedic surgical robots in China are still in their infancy. However, it is worth noting that the Chinese trauma orthopedic robot has made a great breakthrough with the update of image processing technology and navigation tracer technology. The published articles on trauma orthopedic robots are basically from China.^7^ The purpose of this study is to sort out the reports about the application of robots in this field in China and explore its development characteristics and real clinical efficacy. Our research found that the first article reported the clinical application of trauma orthopedic robots since 2005. Subsequently, the relevant articles increased year by year and reached a new peak in 2019. This shows that the research in this field has attracted more and more researchers' attention. Trauma orthopedic robots involved as many as eight kinds of surgery with the increase in application year by year, but the application was mainly focused on the pelvic injury. This was closely related to the complex anatomical structure of the pelvis and the high risk of manual operation.^21^ Especially in the implantation of sacroiliac screw, LC‐II screw, and acetabular anterior and posterior column screw, the “opportunity tunnel” of pelvic fixation was narrow and multiplanar imaging was required to avoid damaging the nearby fragile nerve and vascular structures. Therefore, robots were favored by surgeons because of their precision and minimally invasive characteristics.^22^ This study found that up to 29 articles reported the operation of cannulated screw placement, which also confirmed this phenomenon. At present, there are three kinds of trauma orthopedic robots involved, and the most widely used is “Tianji” orthopedic surgery robot (TiRobot) (Figure 1). This robot is researched and manufactured by Beijing Tinavi Medical Technology Co., Ltd. It is a multi‐indication surgical robot platform suitable for femoral neck, pelvis, and whole spine segments. According to the self‐developed biplane positioning principle, it adopts a unique biplane positioning algorithm to calculate and complete spatial positioning and a modular frame robot to complete path navigation. It has the ability to correct the position offset of screws in bone in real‐time. It uses a six‐degree‐of‐freedom manipulator to expand the effective surgical activity space and tracks the changes in the intraoperative surgical path and patient position in real‐time through the optical tracking system. Finally, the path deviation is corrected through automatic feedback compensation operation to achieve accurate and reliable surgical path positioning.^23^

As for the clinical application effect of trauma orthopedic robots, our research mainly analyzed the outcomes of clinical articles belonging to a cohort study. Wang et al.^24^ and Luo et al.^25^ found that the operation time of the trauma orthopedic robot group was significantly less than that of the traditional group. A total of 10 cohort studies had similar views. However, there were also five cohort studies. Two of them were prospective cohort studies and RCT studies with a high level of evidence.^14^, ^15^ Duan et al.^15^ believed that the total operation duration included the noninvasive period of robot path planning and the invasive period of actual operation. In fact, the operation time from the insertion of the first guide needle to skin closure was very short, which was significantly less than that in the traditional group. At the same time, the operator's operation speed was relatively fast, so most of the time was spent on equipment placement and debugging, image acquisition, and other noninvasive procedures.^15^ This was also the main problem of the trauma orthopedic robot at this stage. The preparation time in the early stage of the operation was too long, which affected the completion speed of the whole operation. Wang et al.^14^ also believed that even if there was little difference in operation time between the two groups, it was an acceptable amount of time for new technology. In addition, the implantation time of the guide needle in the robot‐assisted group was significantly shorter than that in the traditional group, because the number of attempts to insert the guide needle in the robot‐assisted group decreased, and the stable and accurate operation increased. In terms of surgical blood loss, all cohort studies involved believed that the robot group was significantly less than the traditional group, which was closely related to the high precision of the robot and the high success rate of single nail placement.^26^ Similarly, most cohort studies believed that the robot group was significantly reduced in terms of fluoroscopy frequency and fluoroscopy time, which was statistically significant. Only the research of Zhu et al.^16^ found no difference between the two groups. They believed that this was related to the need for additional surgical steps for robotic surgery. In the early days when the system was first introduced, surgeons were not skilled enough. The frequency of intraoperative fluoroscopy was mainly related to the need for repeated confirmation during the placement of the guide needle and cannulated screw, and the TiRobot system also could not help control the insertion depth. Therefore, there was no difference in the above data between the two groups. With the improvement of technology, we hope that the trauma orthopedic robot can make a breakthrough in controlling the preparation time and automatically controlling the insertion depth. In terms of functional score, eight articles believed that there was a statistical difference in functional score between the robot group and the traditional group. However, seven articles believed that there was no statistical difference between the two groups. Therefore, whether the robot group is better than the traditional group in the functional score is still uncertain, which needs to be determined by future research. In terms of bone healing, most studies had found that the robot group was not significantly better than the traditional group.

### 4.1 Study limitation

**Figure 2:**
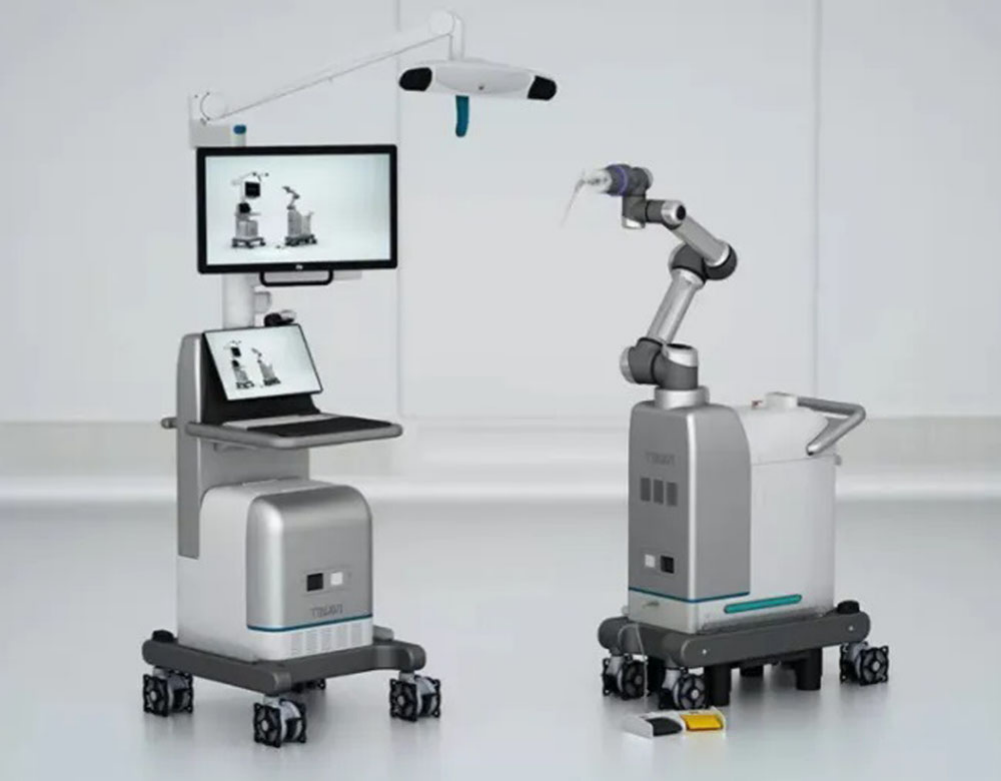
TiRobot

This study is the first systematic review of the clinical application of trauma orthopedic robotic surgery in China. Especially for foreign scholars, there was little information about trauma orthopedic robots in English, which was well supplemented by the results of this study. *Limitations*: 1. The evidence level of the articles included in the study was low, most of which were III–IV. The lack of high‐level studies such as RCT had a certain impact on the results. 2. Some of the included articles included Chinese literature, which was inconvenient for scholars from non‐Chinese countries to further explore (Figure 2).

## 5 CONCLUSION

Robot‐assisted surgery in the clinical application of trauma orthopedics in China is experiencing rapid development. Compared with traditional surgical methods, it has obvious advantages in accuracy, stability, and reducing intraoperative radiation exposure, but there is no final conclusion about functional recovery.

## AUTHOR CONTRIBUTIONS

**Ding Xu**: Conceptualization; funding acquisition; methodology; and writing–original draft. **Weigang Lou**: Formal analysis; and writing–review and editing. **Ming Li**: Methodology. **Jingwei Xiao**: Formal analysis; and writing–original draft. **Hongbao Wu**: Formal analysis; and writing–original draft. **Jianming Chen**: Conceptualization; supervision; and writing–review and editing. All authors have read and approved the final version of the manuscript.

## CONFLICT OF INTEREST

The authors declare no conflict of interest.

## ETHICS STATEMENT

None required.

## TRANSPARENCY STATEMENT

The corresponding author Ding Xu affirms that this manuscript is an honest, accurate, and transparent account of the study being reported; that no important aspects of the study have been omitted; and that any discrepancies from the study as planned (and, if relevant, registered) have been explained.

## Data Availability

The data that support the findings of this study are available from the corresponding author upon reasonable request. Ding Xu had full access to all of the data in this study and takes complete responsibility for the integrity of the data and the accuracy of the data analysis.
